# Integrating Diverse Types of Genomic Data to Identify Genes that Underlie Adverse Pregnancy Phenotypes

**DOI:** 10.1371/journal.pone.0144155

**Published:** 2015-12-07

**Authors:** Jibril Hirbo, Haley Eidem, Antonis Rokas, Patrick Abbot

**Affiliations:** Department of Biological Sciences, Vanderbilt University, Box 35164 Station B, Nashville, TN, 37235–1634, United States of America; Duke University, UNITED STATES

## Abstract

Progress in understanding complex genetic diseases has been bolstered by synthetic approaches that overlay diverse data types and analyses to identify functionally important genes. Pre-term birth (PTB), a major complication of pregnancy, is a leading cause of infant mortality worldwide. A major obstacle in addressing PTB is that the mechanisms controlling parturition and birth timing remain poorly understood. Integrative approaches that overlay datasets derived from comparative genomics with function-derived ones have potential to advance our understanding of the genetics of birth timing, and thus provide insights into the genes that may contribute to PTB. We intersected data from fast evolving coding and non-coding gene regions in the human and primate lineage with data from genes expressed in the placenta, from genes that show enriched expression only in the placenta, as well as from genes that are differentially expressed in four distinct PTB clinical subtypes. A large fraction of genes that are expressed in placenta, and differentially expressed in PTB clinical subtypes (23–34%) are fast evolving, and are associated with functions that include adhesion neurodevelopmental and immune processes. Functional categories of genes that express fast evolution in coding regions differ from those linked to fast evolution in non-coding regions. Finally, there is a surprising lack of overlap between fast evolving genes that are differentially expressed in four PTB clinical subtypes. Integrative approaches, especially those that incorporate evolutionary perspectives, can be successful in identifying potential genetic contributions to complex genetic diseases, such as PTB.

## Introduction

Complex genetic diseases derive from evolutionary and mutational processes that generate segregating variants conferring susceptibility to disease [1–5]. The challenges for identifying disease genes have been well-documented: different approaches traditionally used to identify them can produce large numbers of candidates that explain only modest amounts of variation in risk, and often lack replication [6, 7]. Moreover, the disease itself may constitute a poorly defined or understood phenotype, which means that hypotheses regarding potential contributors to disease are made in the absence of a sufficient understanding of traits in their normal, non-disease states.

One promising approach that has the potential to reduce the number of candidates and increase replication, while identifying broad features of the genotype-to-phenotype map, is the integration of diverse datasets [8–12]. Several integrative approaches have been developed to interrogate the rapidly growing body of high-throughput genomic datasets for identifying genes that may either directly harbor causal variants, or else be involved in complex syndromes [13, 14]. Convergence in identifying disease genes across different data types defines features of the genetic architecture and the functional associations between loci that underlie complex phenotypic traits, and potentially highlight important pathways and gene sets that may be overlooked within the framework of a single data type because of weak effect sizes or because they only indirectly affect the trait of interest [14–18]. Convergence-based approaches have been successful in identifying genetic networks underlying certain cancers, as well as a number of other diseases, including lung, autoimmune and neurodegenerative diseases, type 2 diabetes [15, 17, 18], and even well-studied polygenic traits, such as human height [19].

Pregnancy maintenance and parturition are complex reproductive processes that involve interactions between the fetal, paternal and maternal genomes, and maternal physiology and environment. Complications associated with pre-term birth (PTB) are among the leading causes of mortality worldwide of children under the age of five [20]. PTB is a heterogeneous phenotype that includes nine different obstetrically defined clinical manifestations: infection/inflammation, maternal stress, decidual hemorrhage, uterine distention, cervical insufficiency, placental dysfunction, premature rupture of the membranes, maternal comorbidities, and familial factors [21]. While PTB results from a complex set of causes, various studies have indicated that PTB exhibits moderate heritability [22–27], motivating efforts to identify the genetic factors that confer risk for PTB. Like other human complex genetic traits [28,29], the genetics that characterize PTB most probably involve both coding and non-coding variation at many loci, with causal alleles displaying a range of effect sizes and population frequencies [30–36]. Candidate gene analyses and studies of patterns of differentially expressed genes across various tissues have implicated many variants and numerous differentially expressed genes across various tissues, although few have been replicated or confirmed by genome-wide association studies (GWAS) [37–41]. To date, integrative approaches have not kept pace with the proliferation of new data and data types on PTB, hampering identification of genes and pathways that underlie birth timing (e.g., [41]).

To evaluate the convergence of different data types on PTB, we overlaid datasets that identified fast evolving genes in the human and primate lineage with datasets that identified differentially expressed genes enriched for placental expression across four PTB clinical subtypes. The rationale for this approach follows from the fact that the mechanisms that determine parturition and birth timing in humans are poorly understood [38]. The placenta mediates implantation in pregnancy, performs all of the major organ functions of the developing fetus, and forms the metabolic, immunological and endocrinological interface between mother and fetus [39]. Placental pathologies are a leading cause of diseases of pregnancy, such as pre-eclampsia [40]. Characterizing the genetic features of placentally-expressed genes is thus a necessary step in the effort to understand human parturition and the genetic factors that disrupt pregnancy. Because pregnancy traits have evolved very fast in modern humans, and are obviously closely tied to fitness, the signatures of adaptation and rapid evolution in maternal and fetal traits associated with pregnancy must be reflected in the genes that underlie them [41]. Evolutionary-informed discovery of the genetic contributions to human pregnancy can thus help to pinpoint the genes, functional mechanisms and adaptations that comprise parturition and birth timing in modern humans, and aid in the discovery of genetic elements associated with disease [42].

## Methods

### Gene Expression Data

**Fig 1:**
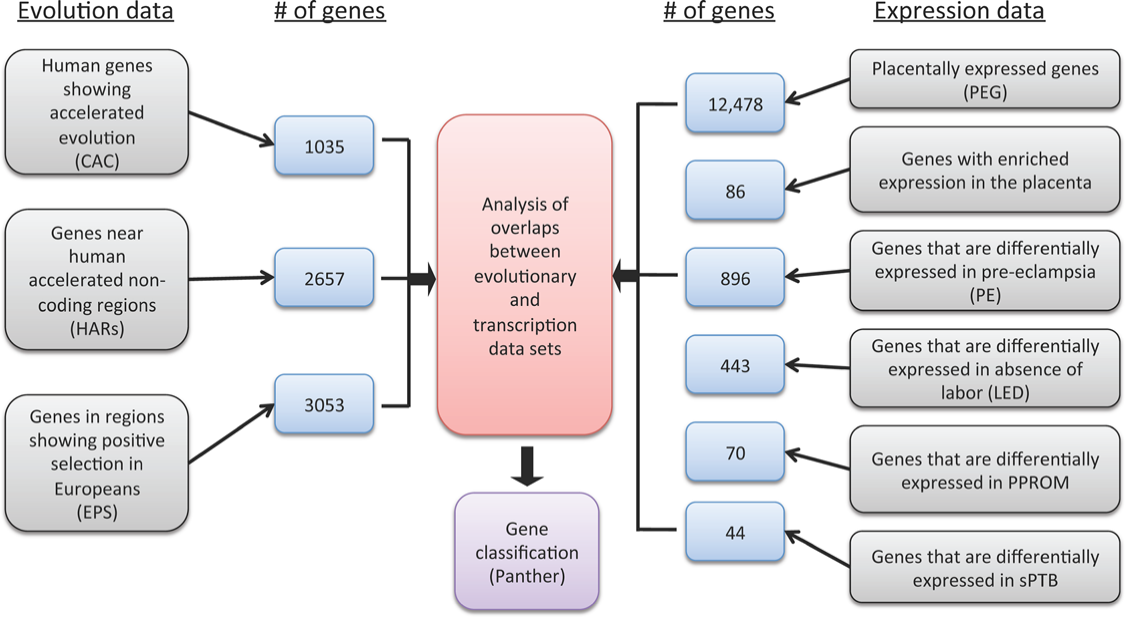
Overview of the scheme for identifying convergence between genes under positive selection and those associated with expression differences in normal pregnancy and various syndromes. Convergence between different data set was determined by overlaying gene sets from each of the data categories using Venn diagram. Genes that fall in overlapping sets were functionally annotated using PANTHER web tool. Key: CAC, Coding Accelerated Changes; EPS, European Positive Selection; HARs, Human Accelerated Regions; PEG, Placental Expressed Genes; PED, Preeclampsia Expressed Differential; LED, Labor Expressed Differential; PPROM, Preterm Premature Rupture of Membranes; sPTB, Spontaneous Preterm Birth.

An overview of the experimental scheme is shown in **Fig 1**. We first downloaded a list of genes expressed in trophoblastic and decidual placenta cells from the Protein Atlas Database (PAD) of a Tissue-Based Map of the Human Proteome ver. 13 [43]. Most of these placentally expressed genes, or PEGs, are expressed in various tissues in addition to the placenta. Only genes that have official gene symbols based on the DAVID gene ID conversion tool were used, and after excluding duplicates, the final list of PEGs from Protein Atlas consisted of 12,478 genes [44, 45]. Next, we downloaded the lists of genes from the PAD that are enriched in placenta (86 genes) and those that are expressed in 23 other tissues. The PAD defines tissue enrichment as those that are expressed at levels at least 5X higher in the focal tissue compared to all other tissues in the body. Our list included those tissues with at least five genes or more that are enriched. Finally, we downloaded lists of differentially expressed genes from four PTB clinical subtypes (preeclampsia (PE), 896 genes; spontaneous or idiopathic preterm birth (sPTB), 44 genes; preterm premature rupture of membranes (PPROM), 70 genes; and presence of birth without labor (Labor Expressed Differentially; LED), 443 genes) compiled from 93 studies (that looked at patients with pregnancy complicated by a particular PTB clinical subtype relative to individuals with normal pregnancies as controls) by Eidem and co-workers [46].

### Evolutionary Data

We also collated three different lists of genes that represent both ancient and more recent signatures of fast evolution in coding and non-coding regions along the human and primate lineage. We used studies that were genome wide, reported lists of genes in the text or as supplemental data, and that captured a range of methods that infer fast evolutionary rates, e.g., site frequency spectrum (SFS), linkage disequilibrium and composite methods (see the **S1 Materials** for explanations of the methods described below). The first list collated data from 11 different studies reporting the 1,035 genes that exhibit signatures of fast evolution in the genic regions among primate lineages based on interspecies comparisons [42, 47–56] (**S1** Table). We call this list Coding Accelerated Changes, or “CAC”. The second list collated data from four studies that identified short elements exhibiting accelerated lineage-specific substitutions in conserved noncoding sequences in vertebrates (known as Human Accelerated Regions or HARs) [57–60]. To generate the lists of 2,657 genes that correspond to 3,939 HARs, we used the Genomic Regions Enrichment of Annotations Tool (GREAT—http://bejerano.stanford.edu/great/public/html/) (**S1** Table) [61]. The third list collated data from 19 studies that analyzed genes in regions associated with signals of positive selection (including genome wide single nucleotide polymorphisms (SNPs), HapMap, HGDP, Perlegen data and sequence data from the 1000 Genomes Project and Complete Genomics in European populations) [62–80]. See the **S1 Materials** for descriptions of analytical methods for measuring selection in human populations, and references therein. We limited our survey to the 3,053 genes in such regions in European populations because most analyses of pregnancy phenotypes are skewed towards individuals of European ancestry. Fast evolving genes identified by this method occurred after emergence of modern humans and out-of-Africa migrations in ancestral European populations. We call this dataset European Positive Selection, or “EPS” (**S1** Table).

In summary, CAC genes therefore correspond to fast evolution in exonic regions (largely determined by ratios of nonsynonymous to synonymous substitutions or dN/dS), and tend to be genes with more ‘ancient’ signatures of fast evolution. In contrast, HARs and EPS genes correspond largely to genes linked to fast evolving non-exonic elements, and constitute genes that tend to be associated with more recent signatures of selection. Below, for simplicity, we generally refer to any genes emerging from these three lists as being “fast evolving”, and we use the terms “coding” CAC genes and “non-coding” HARs and EPS genes to mean the genomic localization of the fast evolving allele, and not the protein coding potential of the genes themselves. Thus, for example, a “fast-evolving EPS gene” is one in the genomic neighborhood of a SNP identified in a scan for accelerated evolution. Possible evolutionary interpretations for genes identified by these different methods are provided in the **S1 Materials**.

### Visualization and Statistical Analysis

CAC, HAR-associated, and EPS genes were overlaid with those from the different gene expression data sets described above. We visualized these data with Venn diagrams using Venny v. 2.0 [81]. We evaluated statistical significance of the overlap between pairs of gene sets by a hypergeometric distribution test as implemented in http://nemates.org/MA/progs/overlap_stats.html [82]. We summarized the overlap using a simple index known as the representation factor (RF), which is the number of overlapping genes divided by the expected number of overlapping genes drawn from two independent groups [82]. An RF > 1 indicates more overlap than expected, whereas an RF < 1 indicates less overlap than expected [82]. We only present significant results for RF > 1, as we are interested in those genes that overlap more than expected by chance. The representation factor (RF) was calculated using the GENCODE ver. 22 estimate of 19,814 genes in the human genome [83].

For the genes we collated, we summarized patterns of biological, molecular, protein and pathway annotations using PANTHER ver. 10.0 (Protein Annotation Through Evolutionary Relationship—http://www.pantherdb.org/) [84]. We evaluated patterns of overrepresentation for overlapping genes in PANTHER, using lists of overlapping genes as tests, and reference lists appropriate to the relevant comparison. For example, for placentally expressed genes derived Protein Atlas, we summarized functional annotations and overrepresentation of those successfully mapped to the ENSEMBL genome archived in the PANTHER database as 2014–4. Alternatively, for analysis of overrepresentation in PANTHER classes of fast evolving genes among all placental genes, our reference gene list was placental genes only, rather than all human genes. Significance was evaluated using a binomial distribution test corrected for multiple tested, as implemented in PANTHER. There were few or no genes that were differentially expressed in sPTB and PPROM that were also fast evolving, probably due to the small numbers of studies that looked at genes differentially expressed in these two PTB clinical subtypes [46]. Therefore, evaluation for overrepresentation was not done for these two phenotypes.

## Results

### The overlap between fast evolving and placentally expressed (PEG) genes

**Fig 2:**
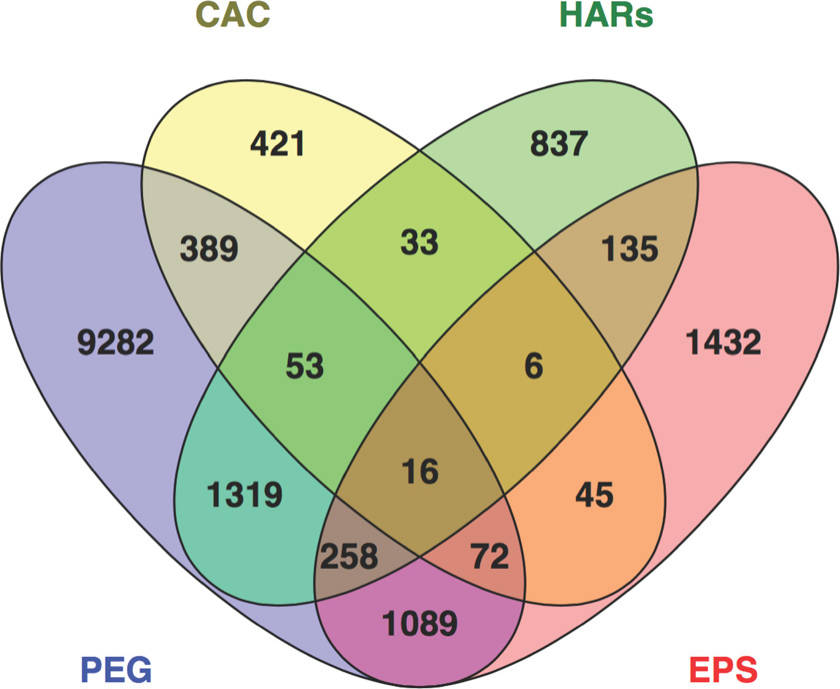
Overlap between genes expressed in placenta and those that have undergone fast evolution.

More than 60% of the protein coding genes in the human genome is expressed in the placenta [43]. Of the 12,478 placental genes we evaluated, however, only 3,196 are fast evolving (about 26% of all placentally expressed genes and about 52% of the 6,106 fast evolving genes that we assembled). There was no evidence that fast evolving genes are overrepresented among all genes expressed in the placenta (hypergeometric test; RF = 0.8; **Fig 2**; **Table 1**). Although we aggregated more HARs and EPS genes, placentally expressed, fast evolving genes are drawn roughly proportionally from coding and non-coding gene sets (about half of each gene list; **Table 1**). Of the 12,478 placentally expressed genes, only 16 genes are classified as fast evolving in each of the three categories (**S1** Table). These include 10 neurodevelopmental genes, namely *AUTS2*, *ASTN2*, *COL25A1*, *GFRA1*, *MGAT5B*, *MTR*, *NFIB*, *PTPRD*, *ROBO1* and *HERC2*, a centrosomal protein gene associated with microcephaly (*CDK5RAP2*), two genes with possible associations with immunity (*FCRL3* and *THSD7B*), a cell adhesion gene associated with epithelial tumorigenesis (*PTPRK*), and a nucleoside transporter (*SLC28A3*).

**Table 1:** Overlap between fast evolving genes and those that exhibit placental expression. Note that some genes occur in more than one category.

Gene categories	All fast evolving genes	Overlap with 12,478 genes expressed in placenta (% of category)
**CAC**	1,035	530 (51.2%)
**HARs**	2,657	1,646 (62.0%)
**EPS**	3,053	1,435 (47.0%)

Placentally expressed genes (PEG) that are fast evolving in at least one category are overrepresented in various biological processes, especially those involving neurological processes, cell adhesion, and various developmental processes, such as mesoderm and nervous systems (**S2** Table). Proteins related to defense, immunity and receptor activity are overrepresented, as are two signaling pathways (epidermal growth factor receptor (EGFR) (*p* value = 0.03) and platelet-derived growth factors (PDGF) (*p* value = 0.04)). Among the most numerous genes in the intersection of fast evolution and placental expression are those involved in Wnt signaling and gonadotropin releasing hormone receptor activity. There are differences between the PEG genes that are in the coding CAC category and the non-coding categories. Coding CAC genes are enriched for genes that code for proteins involved in immune system, including defense/immunity and cytokines, while the placentally expressed, non-coding HARs genes are enriched for genes that encode transcription factors, and proteins involved in development, adhesion and extracellular matrix proteins, and those involved in receptor activity (**S2** Table). Overall, only two pathways are overrepresented among all categories of fast evolution: placental expressed HARs genes are enriched for cadherin and Wnt signaling (p value = 0.003, 0.001) (**S2** Table).

### The overlap between fast evolving genes and genes enriched for placental expression

**Fig 3:**
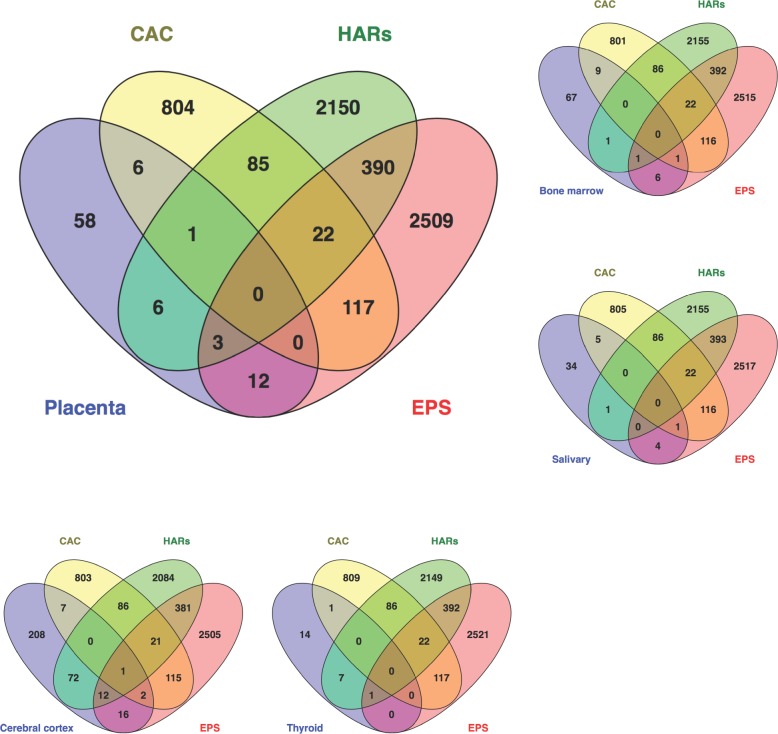
Overlap between the genes enriched in the placenta and those that are fast evolving in the human genome. Similar diagrams are shown for four tissues (cerebral cortex, thyroid, salivary, and bone marrow) that exhibit patterns of significant enrichment of fast evolving genes.

For the genes enriched for tissue expression (expressed 5X more in a given tissue than other tissues), only five of the 24 tissues we evaluated were significantly overrepresented in any of the fast evolution categories, bone marrow, cerebral cortex, placenta, salivary gland and thyroid gland (**Fig 3**; **Table 2**; **S1** Table). Nearly 32.5% of placental enriched genes (30 of 86) are fast evolving genes, more than that all tissues other than thyroid gland (39%) and cerebral cortex (34.6%). The signature of fast evolution differed among the five tissues (**Table 2**). CAC genes tended to be overrepresented among salivary gland (RF = 2.6; *p* value < 0.02), bone marrow (RF = 2.3; *p* value < 0.03) and placentally enriched (RF = 1.6) genes, although the latter was not significant (*p* value < 0.16). By contrast, genes whose expression was enriched in cerebral cortex and thyroid tissues were significantly overrepresented among genes linked to HARs (RF = 2.0; *p* value < 1.6e-10 and RF = 2.6; *p* value < 0.01, respectively). No EPS genes were overrepresented in any tissues. There were no genes in the intersection of placental enrichment and each of the three categories of fast evolution (**Fig 3**). A number of genes overlapped in two categories of fast evolution, however. These include a pregnancy-associated plasma protein A (*PAPPA*), a corticotropin-releasing hormone (*CRH*), a proteoglycan (*EPYC*), and a hepatocyte growth factor (HGF) important in angiogenesis and tumorigenesis (**S1** Table).

**Table 2:** Overlap between fast evolving genes and those that exhibit tissue enrichment in their expression. Only tissues with significant representation factors greater than one are shown, out of 24 tissues evaluated in the Protein Atlas database with more than five enriched genes. Values are genes, with associated representation factors in parentheses and asterisk for values significantly > 1.

Enriched tissues	N	CAC (RF)	HARs (RF)	EPS (RF)	Total unique, fast evolving (%)
**Bone marrow**	85	10 (2.3)*	2	8	18 (21.2)
**Cerebral cortex**	318	10	85 (2.0) **	31	110 (34.6)
**Placenta**	86	7 (1.6)	10	15	28 (32.5)
**Salivary gland**	45	6 (2.6)*	1	5	11 (24.4)
**Thyroid gland**	23	1	8 (2.6) *	1	9 (39.1)
**All genes**	19,814	1,035	2,657	3,053	

* p < 0.05

** p << 0.01.

As with PEGs, those genes enriched for expression in the placenta encode diverse proteins, many of which have catalytic, transport and signaling properties, and are involved in variety of processes typical of placental expression, such as cell adhesion, immunity, proteolysis and hormone biosynthesis (**S3** Table). Probably due to small sample size, most fast evolving, placentally enriched genes are not statistically overrepresented in functional categories. The exception is HARs-associated genes where, relative to all placentally enriched genes, there is overrepresented in adhesion processes (**S3** Table). Among the 30 fast evolving, placentally enriched genes, three (*CGA*, *CGB2*, *CRH*) encode releasing hormones (corticotropin releasing factor receptor signaling, gonadotropin releasing hormone receptor, and thyrotropin releasing hormone receptor, respectively (**S1** Table)). A number of fast evolving, placental genes show tumorigenic or tumor suppression activity (*ADAM12*, *ADAMTS18*, *CAPN6*, *EGFL6*, *HTRA4*, *LIN28B*) and others are involved in disorders associated with epithelial and connective tissues (*FBN2*) or have immune functions (*IL1RL1*, *PRG2*, *SIGLEC6*). Three members of the pappalsin family are fast evolving in the placenta (*PAPPA*, *PAPPA2*, *PAPPA-AS1*), and altogether, seven members of the pregnancy-specific glycoproteins are fast evolving (**S1** Table).

### The overlap between fast evolving genes and differentially expressed genes in PTB clinical subtypes

**Fig 4:**
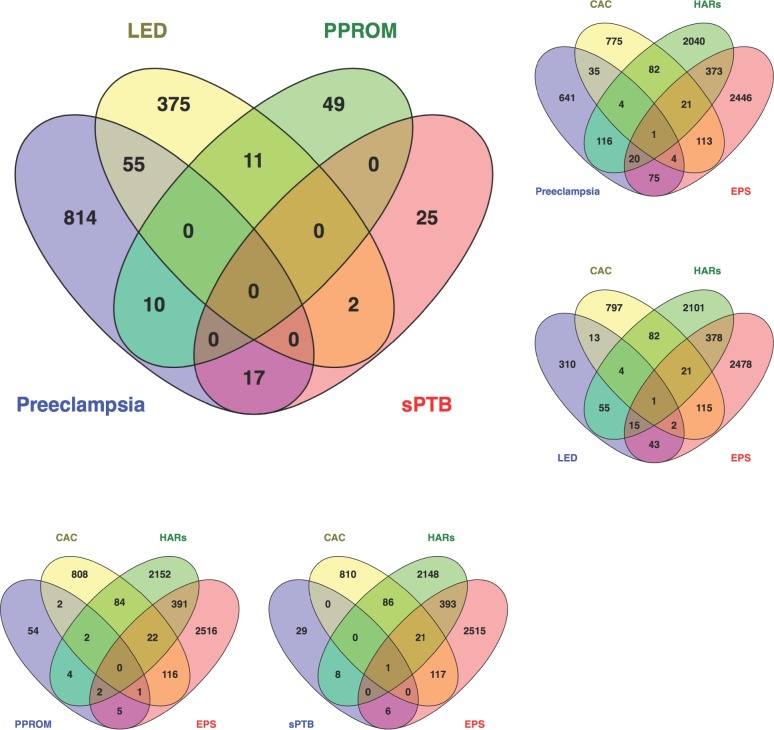
Overlap between genes differentially expressed in four PTB clinical subtypes (large diagram; collated by Eidem et al. 2015), and the overlaps between each of these clinical subtypes and fast evolving genes.

Of those genes differentially expressed in the PTB clinical subtypes, the proportion of fast evolving genes ranges from 23% to 34%, with sPTB having the largest fraction of fast evolving genes (**Table 3**). The large fraction of fast evolving sPTB genes is largely driven by fact that 8 of 44 genes differentially expressed in sPTB are linked to HARs, although this overrepresentation was not statistically significant (sPTB; hypergeometric test; RF = 1.1; *p* value = 0.37, **Table 3**). No fast evolving genes were common to the four categories of differentially expressed PTB clinical subtypes, nor did any genes in the four PTB clinical subtypes overlap in each of the three categories of fast evolution (**Fig 4**), perhaps reflecting real underlying differences in the biological axes categorizing these clinical subtypes and the breadth and complexity of the phenotypes subsumed under the various clinical subtype categories. For example, PPROM and sPTB share no fast evolving genes in common. Nevertheless, although sample sizes are small, 14% and 39% of the PPROM and sPTB differential expressed genes overlapped with those in differentially expressed in PE, highlighting biological commonalities both of these clinical subtypes likely share with PE.

**Table 3:** Overlap between fast evolving genes and those that show differential expression in PTB clinical subtypes (preeclampsia, birth without labor (LED), premature rupture of membranes (PPROM), and spontaneous pre-term birth (sPTB). RF factors greater than 1.0 are indicated in parentheses, and asterisks indicates statistical significance.

	N	Genes with fast evolution in coding regions in interspecies comparisons (CAC)	Genes linked to human accelerated regions (HARs)	Genes linked to fast evolution in European populations (EPS)	Total, unique fast evolving genes (%)
Preeclampsia	896	44	141 (1.2)*	100	255 (28.5)
LED	443	20	75 (1.3)*	61	133 (30.0; 1.0)
PPROM	70	5 (1.4)	8	8	16 (22.8)
sPTB	44	1	9 (1.6)	7 (1.0)	15 (34.1; 1.1)

* p < 0.01.

In terms of their functional annotations, no fast evolving PTB clinical subtype genes are overrepresented in PANTHER categories (**S4** Table). However, these genes fall in categories that are consistent with recognized disease pathways in pregnancy, including the P53, 5-hydroxytreptamine (serotonin or 5-HT) degradation, and TGF-β signaling pathways, and various pathways involved in neurodevelopment and immune system processes. As was the case with placental enriched, fast evolving genes, a number of fast evolving genes that are differentially expressed in PTB clinical subtypes express tumor-proliferative or suppressive activity. These included *WWOX* (common to preeclampsia, HARs and EPS), a gene that play a role in apoptosis and act as tumor suppressor [85, 86]. *KDR* (common to LED, HARs and EPS) is a gene involved in mediating endothelial proliferation, survival, migration, tubular morphogenesis and sprouting [87, 88]. Also common to that group is *ITPR1*, which is a gene that mediates calcium release from the endoplasmic reticulum and triggers apoptosis [89, 90]. As well, two of the three genes play roles in neurogenesis [91–95], while *KDR* has been implicated in recurrent pregnancy loss [96]. A number of genes that are differentially expressed in PTB clinical subtypes and fast evolving are immune related or involved in angiogenesis, such as *CFB* which is involved in complement activation, and *CXCR4*, which is a chemokine receptor. Finally, two genes differentially expressed in PTB clinical subtypes and classified as fast evolving in each of the three categories, NFIB (preeclampsia and LED) and *CXCR4 (*sPTB) (**S1** Table).

## Discussion

Common heritable diseases are evolutionary conundrums. Debates about disease models that can account for alleles that segregate at appreciable frequencies hinge on population genetic assumptions about evolutionary history, effect sizes, and demography, and whether the loci that underlie diseases ultimately will conform to modeling constraints [6, 7, 97]. One alternative method that has emerged in recent years is a “nonparametric” approach, which is based on the assumption that different data types can converge on the loci underlying common diseases, even if the data do not readily conform to contemporary disease categories or disease models.

In this study we asked, to what extent do placental gene sets derived from evolutionary-based analyses converge on those derived from expression analyses, and what light can the intersection of such data shed on our understanding of the genetic basis of PTB clinical subtypes? We found that when fast evolving genes and elements are aggregated by evolutionary rate variation in coding and non-coding regions and partitioned by differential expression in the placenta, they converge on a small number of genes that may be candidates for PTB. For example, fast-evolving PEG genes typically encode membrane-bound proteins with functions related to binding and signaling between cells and the extracellular matrix. Disruption of membrane formation and rupture are well-characterized pathologies of normal pregnancy [98]. Likewise, of the genes that are enriched for placental expression, nearly 35% are fast evolving, greater than all but two of the other tissues we evaluated (thyroid gland and cerebral cortex). Many of these placental genes are evolving rapidly in coding regions. By comparison, the fast evolving genes expressed in the brain tend to be associated with HARs, possibly reflecting fundamental differences in how selection has acted on the human brain and placenta [99]. A third of these placentally enriched fast evolving genes have well-characterized roles in pregnancy and differentially expressed in PTB clinical subtypes (*EPYC*, *HGF*, *PSG2*, *PSG3*, *PSG4*, *CRH*, *PAPPA*, *PSG1*, *PSG5*, *PSG11*), and as with fast evolving PEG genes, are signaling or extracellular molecules with roles in inflammation, neurodevelopment, and inflammation. Despite performing pregnancy related functions, most of the 16 PEG genes that exhibit pattern of fast evolution in all selection categories (10/16) were not differentially expressed in PTB clinical subtypes. Thus dysregulation in these genes might contribute to pregnancy pathologies by processes that do not involve expression modulation.

### Pathways enriched in PEG and fast evolving PEG genes

In terms of pathways in which they are expressed, dysregulation of any of the genes uniquely enriched for placental expression will conceivably underlie pregnancy related pathologies. Relative to all genes in the human genome, PEG genes are enriched for two pathways (EGF and PDGF signaling). Fast-evolving PEG genes (those associated with HARs) are enriched for two additional pathways (cadherin and Wnt signaling). Interestingly, all four pathways play critical roles in regulating growth, proliferation and differentiation of mammalian cells [100, 101], roles that are indispensable for normal development and functioning of the placenta. In addition, EGFR signaling has been shown to stimulate angiogenesis, promote cytotrophoblast migration and invasion, and block apoptosis [102, 103], while PDGF is important in regulating trophoblast angiogenesis [104, 105]. The Wnt signaling pathway is facilitator of cell-cell signaling events during embryogenesis [106, 107], and plays several roles in human placentation [107, 108]. Cadherins are a group of transmembrane glycoproteins involved in cell adhesion and tissue formation [109–113]. Most of the genes from the cadherin superfamily are expressed in the embryonic and adult nervous system and have been implicated in diseases of the central nervous system. Wnt and cadherin signaling share a key component that facilitates normal cascades within both pathways, and several studies have shown cross talk between the two [114–117]. Overall, the four pathways are crucial for successful implantation and development of early pregnancy [118, 119]. Furthermore, dysregulation of the EGFR, PDGF and Wnt genes have been implicated in several pregnancy pathologies: complete hydratidiform mole (a rare mass or growth that forms inside the uterus at the beginning of a pregnancy), low birth weight, intrauterine growth restriction (IUGR), recurrent abortions and PE [108, 120–126].

### Fast evolving genes of interest in PTB clinical subtypes

While there was no functional enrichment of fast evolving genes among genes differentially expressed between preeclampsia or birth with labor and normal or birth without labor, respectively, a number of genetic pathways differs between fast evolving PE and LED genes (**S1** Table), possibly highlighting contrasting axes along which these two clinical subtypes segregate (there were too few PPROM and sPTB fast evolving genes for meaningful comparisons). For example, genes in the TGFβ signaling and 5-hydroxytryptamine degradation pathways are fast evolving in coding regions in PE, but not in LED. 5-HT is thought to interfere with the hormonal mechanisms responsible for the maintenance of gestation by hindering the production of progesterone needed for the maintenance of gestation in mice [127]. It is thought that many crucial fetal neurodevelopmental processes are regulated by 5-HT, both from the maternal system early in development and later from the fetal system. 5-HT has been shown to induce labor by stimulating contraction of human uterine smooth muscle myometrium through special contractile receptors expressed in pregnant human myometrium [128, 129]. The contractile effects by 5-HT in myometrium have been identified in species that have discoidal placental types like rabbit, rat and guinea pig, while in contrast, 5-HT inhibits myometrium contractions of a species with diffuse placental types like pig [129, 130]. Thus, 5-HT may have played an important role in the differentiation of placental forms. The TGFβ signaling pathway, an evolutionarily conserved pathway that plays a fundamental role in cell growth and differentiation [131], has been implicated in regulating vascular endothelia growth factors that have been shown to underlie PE [132]. The genes in the TGFβ pathway play roles in preparation of the endometrium for implantation, embryo development and pregnancy [133]. Furthermore, TGFβ is an angiogenetic factor, and variants in several angiogenetic factors such as eNOS and FLT1 have been implicated in PE [35]. That we identified genes the TGFβ signaling pathway supports the view that PE may be a pathological legacy of the human pattern of interstitial implantation [52, 134, 135]. Recent work has suggested that there might be convergence of genetic factors that underlie placental diseases like PE and larger evolutionary patterns in placental traits in mammals [136]. If so, genes involved in mechanisms that distinguish different placentation types and placental phenotypes in mammalian species are prime candidates for involvement in human pregnancy pathologies.

### The evolutionary framework for discovering genes underlying pregnancy related phenotypes in humans

Human have evolved a distinct ensemble of traits relative to our close primate relatives. The four most cited ones, bipedalism, large brain size, metabolism and immune system have been used to formulate hypotheses to explain unique features of human pregnancy [137–145]. From a clinical perspective, the goal is to understand both unique and shared features of gestation timing in humans (normally ~38–42 weeks and vary by up to 37 days), and the mechanisms/pathways that underlie these traits [146]. The central issue is that the genetics that underlie such traits as bipedalism have yet to be discovered, but may provide crucial insights into human pregnancy. For example, genome-wide scans of individuals that suffer from Unertan syndrome, a rare quadrupedal gait phenotype, implicated the *VLDLR* gene which encodes the very low-density lipoprotein receptor, a component of the *Reelin* signaling pathway involved in neuroblast migration in the cerebral cortex and cerebellum [147]. Interestingly, this gene is moderately and highly expressed in normal trophoblastic and decidual cells, respectively [43], and is linked to HARs. Moreover, EPS genes that are expressed in the placenta are enriched for categories such as anatomical structure morphogenesis, and two of three genes from TGF-β signaling pathway identified in genes differential expressed in PE cases are involved in bone formation. Thus, an evolutionary perspective on identifying genes involved in pregnancy pathologies also broadens the scope for understanding the genetics of bipedalism.

Finally, the human brain has undergone rapid evolution, and the genes involved in brain development and their regulatory elements exhibit strong patterns of accelerated evolution [99, 148–151]. The majority of fast evolving, placentally enriched genes (18 of 30) have neurodevelopmental functions, and a number of fast evolving genes in overlapping gene sets is in pathways that either perform brain related functions or have been implicated in diseases of the central nervous system (**S5** Table). Three fast evolving genes (NBPF11, NBPF12 and NBPF15) that are differentially expressed in both sPTB and PE are part of a neuroblastoma breakpoint (NBPF) gene family that has been shown to exhibit neuron-specific expression and copy number variations. These NBPF genes have been implicated in both evolutionary and contemporary variation in brain size among primate and human lineages, and an array of pathologies of the central nervous system, including microcephaly, macrocephaly, autism, schizophrenia and mental retardation [152–159]. Coupling these results regarding the developmental genes with the fact that genes involved in inflammatory/immune response and membrane homeostasis also emerged in our study, a general implication is that evolutionary history has potential to not only inform our understanding of pregnancy pathologies, but also generate hypotheses regarding how changes in neurogenesis, immunity, and membranes have influenced the evolution of human pregnancy.

## Conclusions

Changes in our evolutionary past might have made us susceptible to some pathologies of pregnancy. Despite numerous studies on the genetics of pregnancy and its many diseases and syndromes, our understanding of the genetic factors at play remains incomplete and biased towards much-studied genes that generally underlie feto-maternal interaction in anti/pro-inflammatory pathways [160]. This study highlight both the extent to which there is limited integration of disparate pregnancy related genetic data, and the promise of such integration. Integrative approaches such as these, especially those that incorporate evolutionary, comparative perspectives can be successful in identifying promising avenues for research on complex heritable diseases that have emerged out of the unique changes in our evolutionary past.

## Supporting Information

S1 Materials Additional methods and rationale for detecting overlap of regions and genes exhibiting accelerated evolutionary rates. (DOCX)

S1 Table Lists of genes used for all of the analyses described. (XLSX)

S2 Table Panther categorization of the 12,248 placental expressed genes and their overlap with fast evolving genes. (XLSX)

S3 Table Panther categorization of the placental enriched genes from the Protein Atlas Database and their overlap with fast evolving genes. (XLSX)

S4 Table Panther categorization of the genes differentially expressed in four PTB clinical subtypes (collated by Eidem et al. 2015) and their overlap with fast evolving genes. There were no overrepresented fast evolving genes in any category, and thus these tables are not shown.
(XLSX)

S5 Table Summary of selected genes that exhibit the overlap across multiple categories of fast evolution and expression or disease phenotypes, and synopses of Entrez gene summaries and associated phenotypes. See Table for the full list of overlapping genes.
(DOCX)

S6 Table A: Overlap between genes that fall in genomic regions that are fast evolving in European populations based on different selection methods on 1000 Genomes project data. There is proportionally more overlap between data from STR method and site frequency spectrum. B: Overlap between fast evolving genes in European populations based on integrated haplotype scores (iHS) methods on four different data type of European populations. There is proportionally more overlap between genes inferred from HapMapII and human genome diversity project data than any other pairwise comparison. Statistical significance of the overlap between genes from different methods inferred using hypergeometric method as implemented in http://nemates.org/MA/progs/overlap_stats.html.
(DOCX)

## References

[1] Maxwell EK, Schnitzler CE, Havlak P, Putnam NH, Nguyen A-D, Moreland RT, et al Evolutionary profiling reveals the heterogeneous origins of classes of human disease genes. implications for modeling disease genetics in animals. BMC Evol Biol 2014, 14:212 10.1186/s12862-014-0212-1 25281000 PMC4219131

[2] Domazet-Lošo T, Tautz D. An ancient evolutionary origin of genes associated with human genetic diseases. Mol Biol Evol 2008, 25:2699–2707. 10.1093/molbev/msn214 18820252 PMC2582983

[3] Cai JJ, Borenstein E, Chen R, Petrov DA. Similarly strong purifying selection acts on human disease genes of all evolutionary ages. Genome Biol Evol 2009, 1:131–144. 10.1093/gbe/evp013 20333184 PMC2817408

[4] López‐Bigas N, Ouzounis CA. Genome‐wide identification of genes likely to be involved in human genetic disease. Nucleic Acids Res 2004, 32:3108–3114. 15181176 10.1093/nar/gkh605 PMC434425

[5] Dickerson JE, Robertson DL. On the origins of Mendelian disease genes in man. the impact of gene duplication. Mol Biol Evol 2012, 29:2284–2284. 10.1093/molbev/msr111 PMC3709195 21705381

[6] Mitchell K. What is complex about complex disorders? Genome Biol 2012, 13:237 10.1186/gb-2012-13-1-237 22269335 PMC3334577

[7] Gibson G. Rare and common variants. twenty arguments. Nat Rev Genet 2012, 13:135–145. 10.1038/nrg3118 22251874 PMC4408201

[8] Chen R, Morgan A, Dudley J, Deshpande T, Li L, Kodama K, et al FitSNPs: highly differentially expressed genes are more likely to have variants associated with disease. Genome Biol 2008, 9:R170 10.1186/gb-2008-9-12-r170 19061490 PMC2646274

[9] Wang L, Jia P, Wolfinger RD, Chen X, Zhao Z. Gene set analysis of genome-wide association studies. Methodological issues and perspectives. Genomics 2011, 98:1–8. 10.1016/j.ygeno.2011.04.006 21565265 PMC3852939

[10] Edwards SL, Beesley J, French JD, Dunning AM. Beyond GWASs. illuminating the dark road from association to function. Am J Hum Genet 2013, 93:779–797. 10.1016/j.ajhg.2013.10.012 24210251 PMC3824120

[11] Robinson MR, Wray NR, Visscher PM. Explaining additional genetic variation in complex traits. Trends Genet 2014, 30:124–132. 10.1016/j.tig.2014.02.003 24629526 PMC4639398

[12] Ritchie MD, Holzinger ER, Li R, Pendergrass SA, Kim D. Methods of integrating data to uncover genotype-phenotype interactions. Nat Rev Genet 2015, 16:85–97. 10.1038/nrg3868 25582081

[13] Schadt EE, Lamb J, Yang X, Zhu J, Edwards S, GuhaThakurta D, et al An integrative genomics approach to infer causal associations between gene expression and disease. Nat Genet 2005, 37:710–717. 15965475 10.1038/ng1589 PMC2841396

[14] Ramanan VK, Shen L, Moore JH, Saykin AJ. Pathway analysis of genomic data: Concepts, methods, and prospects for future development. Trends Genet 2012, 28:323–332. 10.1016/j.tig.2012.03.004 22480918 PMC3378813

[15] Xiong Q, Ancona N, Hauser ER, Mukherjee S, Furey TS. Integrating genetic and gene expression evidence into genome-wide association analysis of gene sets. Genome Res 2012, 22:386–397. 10.1101/gr.124370.111 21940837 PMC3266045

[16] Talwar P, Silla Y, Grover S, Gupta M, Agarwal R, Kushwaha S, et al Genomic convergence and network analysis approach to identify candidate genes in Alzheimer’s disease. BMC Genomics 2014, 15:199–199. 10.1186/1471-2164-15-199 24628925 PMC4028079

[17] Moffatt MF, Kabesch M, Liang L, Dixon AL, Strachan D, Heath S, et al Genetic variants regulating ORMDL3 expression contribute to the risk of childhood asthma. Nature 2007, 448:470–473. 17611496 10.1038/nature06014

[18] Zhong H, Yang X, Kaplan LM, Molony C, Schadt EE. Integrating pathway analysis and genetics of gene expression for genome-wide association studies. Am J Hum Genet, 86:581–591. 10.1016/j.ajhg.2010.02.020 20346437 PMC2850442

[19] Lui JC, Nilsson O, Chan Y, Palmer CD, Andrade AC, Hirschhorn JN, et al Synthesizing genome-wide association studies and expression microarray reveals novel genes that act in the human growth plate to modulate height. Hum Mol Genet 2012, 21:5193–5201. 10.1093/hmg/dds347 22914739 PMC3490510

[20] Liu L, Oza S, Hogan D, Perin J, Rudan I, Lawn JE, et al Global, regional, and national causes of child mortality in 2000–13, with projections to inform post-2015 priorities. an updated systematic analysis. The Lancet, 385:430–440. 10.1016/S0140-6736(14)61698-6 25280870

[21] Manuck TA, Esplin MS, Biggio J, Bukowski R, Parry S, Zhang H, et al The phenotype of spontaneous preterm birth. application of a clinical phenotyping tool. Am J Obstet Gynecol 2015, 212:487.e1–487.e11. 25687564 10.1016/j.ajog.2015.02.010 PMC4456184

[22] Lunde A, Melve KK, Gjessing HK, Skjaerven R, Irgens LM. Genetic and environmental influences on birth weight, birth length, head circumference, and gestational age by use of population-based parent-offspring data. Am J Epidemiol 2007, 165:734–41. 17311798 10.1093/aje/kwk107

[23] York TP, Eaves LJ, Lichtenstein P, Neale MC, Svensson A, Latendresse S, et al Fetal and maternal genes’ influence on gestational age in a quantitative genetic analysis of 244,000 Swedish births. Am J Epidemiol 2013, 178:543–50. 10.1093/aje/kwt005 23568591 PMC3736752

[24] York TP, Strauss JF 3rd, Neale MC, Eaves LJ. Estimating fetal and maternal genetic contributions to premature birth from multiparous pregnancy histories of twins using MCMC and maximum-likelihood approaches. Twin Res Hum Genet 2009, 12:333–42. 10.1375/twin.12.4.333 19653833 PMC2913409

[25] Svensson AC, Sandin S, Cnattingius S, Reilly M, Pawitan Y, Hultman CM, et al Maternal effects for preterm birth. a genetic epidemiologic study of 630,000 families. Am J Epidemiol 2009, 170:1365–72. 10.1093/aje/kwp328 19854802

[26] Wilcox AJ, Skjaerven R, Lie RT. Familial patterns of preterm delivery: Maternal and fetal contributions. Am J Epidemiol 2008, 167:474–9. 18048376 10.1093/aje/kwm319

[27] Boyd HA, Poulsen G, Wohlfahrt J, Murray JC, Feenstra B, Melbye M. Maternal contributions to preterm delivery. Am J Epidemiol 2009, 170:1358–64. 10.1093/aje/kwp324 19854807 PMC2800264

[28] Gudbjartsson DF, Walters GB, Thorleifsson G, Stefansson H, Halldorsson BV, Zusmanovich P, et al Many sequence variants affecting diversity of adult human height. Nat Genet 2008, 40:609–615. 10.1038/ng.122 18391951

[29] Lettre G, Jackson AU, Gieger C, Schumacher FR, Berndt SI, Sanna S, et al Identification of ten loci associated with height highlights new biological pathways in human growth. Nat Genet 2008, 40:584–591. 10.1038/ng.125 18391950 PMC2687076

[30] Weedon MN, Lango H, Lindgren CM, Wallace C, Evans DM, Mangino M, et al Genome-wide association analysis identifies 20 loci that influence adult height. Nat Genet 2008, 40:575–583. 10.1038/ng.121 18391952 PMC2681221

[31] Aulchenko YS, Struchalin MV, Belonogova NM, Axenovich TI, Weedon MN, Hofman A, et al Predicting human height by Victorian and genomic methods. Eur J Hum Genet 2009, 17:1070–1075. 10.1038/ejhg.2009.5 19223933 PMC2986552

[32] Lango Allen H, Estrada K, Lettre G, Berndt SI, Weedon MN, Rivadeneira F, et al Hundreds of variants clustered in genomic loci and biological pathways affect human height. Nature 2010, 467:832–838. 10.1038/nature09410 20881960 PMC2955183

[33] Wood AR, Esko T, Yang J, Vedantam S, Pers TH, Gustafsson S, et al Defining the role of common variation in the genomic and biological architecture of adult human height. Nat Genet 2014, 46:1173–1186. 25282103 10.1038/ng.3097 PMC4250049

[34] Johnson MP, Brennecke SP, East CE, Göring HHH, Kent JW Jr, Dyer TD, et al Genome-wide association scan identifies a risk locus for preeclampsia on 2q14, near the inhibin, beta B Gene. PLoS ONE 2012, 7:e33666 10.1371/journal.pone.0033666 22432041 PMC3303857

[35] Tuteja G, Cheng E, Papadakis H, Bejerano G. PESNPdb. A comprehensive database of SNPs studied in association with pre-eclampsia. Placenta 2012, 33:1055–1057. 10.1016/j.placenta.2012.09.016 23084601

[36] Uzun A, Laliberte A, Parker J, Andrew C, Winterrowd E, Sharma S, et al dbPTB. a database for preterm birth. Database 2012, 2012. 10.1093/database/bar069 PMC3275764 22323062

[37] Wu W, Clark E, Manuck T, Esplin M, Varner M, Jorde L. A genome-wide association study of spontaneous preterm birth in a European population. F1000Research 2013, 2.

[38] Swaggart KA, Pavlicev M, Muglia LJ. Genomics of preterm birth. Cold Spring Harb Perspect Med 2015, 5. 10.1101/cshperspect.a023127 PMC4315919 25646385

[39] Burton GJ, Jauniaux E. What is the placenta? Am J Obstet Gynecol 2015, 213:S6.e1–S6.e4. 26428504 10.1016/j.ajog.2015.07.050

[40] Cross JC. Placental function in development and disease. Reprod Fertil Dev 2005, 18:71–76. 10.1071/rd05121 16478604

[41] Brown EA, Ruvolo M, Sabeti PC. Many ways to die, one way to arrive: How selection acts through pregnancy. Hum Genet 2013, 29:585–592. 10.1016/j.tig.2013.03.001 23566676

[42] Plunkett J, Doniger S, Orabona G, Morgan T, Haataja R, Hallman M, et al An evolutionary genomic approach to identify genes involved in human birth timing. PLoS Genet 2011, 7:e1001365 10.1371/journal.pgen.1001365 21533219 PMC3077368

[43] Uhlén M, Fagerberg L, Hallström BM, Lindskog C, Oksvold P, Mardinoglu A, et al Tissue-based map of the human proteome. Science 2015, 347. 10.1126/science.1260419 25613900

[44] Huang DW, Sherman BT, Lempicki RA. Systematic and integrative analysis of large gene lists using DAVID bioinformatics resources. Nat Protoc 2008, 4:44–57. 10.1038/nprot.2008.211 19131956

[45] Huang DW, Sherman BT, Lempicki RA. Bioinformatics enrichment tools. paths toward the comprehensive functional analysis of large gene lists. Nucleic Acids Res 2009, 37:1–13. 10.1093/nar/gkn923 19033363 PMC2615629

[46] Eidem HR, Ackerman WE, McGary KL, Abbot P, Rokas A. Gestational tissue transcriptomics in term and preterm human pregnancies: A systematic review and meta-analysis. BMC Med Genomics 2015, 8:27 10.1186/s12920-015-0099-8 26044726 PMC4456776

[47] Bustamante CD, Fledel-Alon A, Williamson S, Nielsen R, Todd Hubisz M, Glanowski S, et al Natural selection on protein-coding genes in the human genome. Nature 2005, 437:1153–1157. 16237444 10.1038/nature04240

[48] Bakewell MA, Shi P, Zhang J. More genes underwent positive selection in chimpanzee evolution than in human evolution. Proc Natl Acad Sci 2007, 104:7489–7494. 17449636 10.1073/pnas.0701705104 PMC1863478

[49] Gibbs RA, Rogers J, Katze MG, Bumgarner R, Weinstock GM, Mardis ER, et al Evolutionary and biomedical insights from the rhesus macaque genome. Science 2007, 316:222–234. 17431167 10.1126/science.1139247

[50] Enard D, Depaulis F, Roest Crollius H. Human and non-human primate genomes share hotspots of positive selection. PLoS Genet 2010, 6:e1000840 10.1371/journal.pgen.1000840 20140238 PMC2816677

[51] Crisci JL, Wong A, Good JM, Jensen JD. On characterizing adaptive events unique to modern humans. Genome Biol Evol 2011, 3:791–798. 10.1093/gbe/evr075 21803765 PMC3163466

[52] Crosley EJ, Elliot MG, Christians JK, Crespi BJ. Placental invasion, preeclampsia risk and adaptive molecular evolution at the origin of the great apes. evidence from genome-wide analyses. Placenta 2013, 34:127–32. 10.1016/j.placenta.2012.12.001 23266291

[53] Gaya-Vidal M, Alba M. Uncovering adaptive evolution in the human lineage. BMC Genomics 2014, 15:599 10.1186/1471-2164-15-599 25030307 PMC4124166

[54] Worley et al The common marmoset genome provides insight into primate biology and evolution. Nat Genet 2014, 46:850–857. 10.1038/ng.3042 25038751 PMC4138798

[55] Nielsen R, Bustamante C, Clark AG, Glanowski S, Sackton TB, Hubisz MJ, et al A scan for positively selected genes in the genomes of humans and chimpanzees. PLoS Biol 2005, 3:e170 15869325 10.1371/journal.pbio.0030170 PMC1088278

[56] George RD, McVicker G, Diederich R, Ng SB, MacKenzie AP, Swanson WJ, et al Trans genomic capture and sequencing of primate exomes reveals new targets of positive selection. Genome Res 2011, 21:1686–1694. 10.1101/gr.121327.111 21795384 PMC3202285

[57] Pollard KS, Salama SR, King B, Kern AD, Dreszer T, Katzman S, et al Forces shaping the fastest evolving regions in the human genome. PLoS Genet 2006, 2:e168 17040131 10.1371/journal.pgen.0020168 PMC1599772

[58] Prabhakar S, Noonan JP, Pääbo S, Rubin EM. Accelerated evolution of conserved noncoding sequences in humans. Science 2006, 314:786–786. 17082449 10.1126/science.1130738

[59] Bird C, Stranger B, Liu M, Thomas D, Ingle C, Beazley C, et al Fast-evolving noncoding sequences in the human genome. Genome Biol 2007, 8:R118 17578567 10.1186/gb-2007-8-6-r118 PMC2394770

[60] Bush E, Lahn B. A genome-wide screen for noncoding elements important in primate evolution. BMC Evol Biol 2008, 8:17 10.1186/1471-2148-8-17 18215302 PMC2242780

[61] McLean CY, Bristor D, Hiller M, Clarke SL, Schaar BT, Lowe CB, et al GREAT improves functional interpretation of cis-regulatory regions. Nat Biotech 2010, 28:495–501. 10.1038/nbt.1630 PMC4840234 20436461

[62] Carlson CS, Thomas DJ, Eberle MA, Swanson JE, Livingston RJ, Rieder MJ, et al Genomic regions exhibiting positive selection identified from dense genotype data. Genome Res 2005, 15:1553–1565. 16251465 10.1101/gr.4326505 PMC1310643

[63] Hinds DA, Stuve LL, Nilsen GB, Halperin E, Eskin E, Ballinger DG, et al Whole-genome patterns of common DNA variation in three human populations. Science 2005, 307:1072–1079. 15718463 10.1126/science.1105436

[64] Frazer et al 2007. A second generation human haplotype map of over 3.1 million SNPs. Nature 2007, 449:851–861. 17943122 10.1038/nature06258 PMC2689609

[65] Kimura R, Fujimoto A, Tokunaga K, Ohashi J. A practical genome scan for population-specific strong selective sweeps that have reached fixation. PLoS ONE 2007, 2:e286 17356696 10.1371/journal.pone.0000286 PMC1805687

[66] Sabeti PC, Varilly P, Fry B, Lohmueller J, Hostetter E, Cotsapas C, et al Genome-wide detection and characterization of positive selection in human populations. Nature 2007, 449:913–918. 17943131 10.1038/nature06250 PMC2687721

[67] Tang K, Thornton KR, Stoneking M. A new approach for using genome scans to detect recent positive selection in the human genome. PLoS Biol 2007, 5:e171 17579516 10.1371/journal.pbio.0050171 PMC1892573

[68] Williamson SH, Hubisz MJ, Clark AG, Payseur BA, Bustamante CD, Nielsen R. Localizing recent adaptive evolution in the human genome. PLoS Genet 2007, 3:e90 17542651 10.1371/journal.pgen.0030090 PMC1885279

[69] Barreiro LB, Laval G, Quach H, Patin E, Quintana-Murci L. Natural selection has driven population differentiation in modern humans. Nat Genet 2008, 40:340–345. 10.1038/ng.78 18246066

[70] Johansson Å, Gyllensten U. Identification of local selective sweeps in human populations since the exodus from Africa. Hereditas 2008, 145:126–137. 10.1111/j.0018-0661.2008.02054.x 18667002

[71] Pickrell JK, Coop G, Novembre J, Kudaravalli S, Li JZ, Absher D, et al Signals of recent positive selection in a worldwide sample of human populations. Genome Res 2009, 19:826–837. 10.1101/gr.087577.108 19307593 PMC2675971

[72] Chen H, Patterson N, Reich D. Population differentiation as a test for selective sweeps. Genome Res 2010, 20:393–402. 10.1101/gr.100545.109 20086244 PMC2840981

[73] Grossman SR, Shlyakhter I, Karlsson EK, Byrne EH, Morales S, Frieden G, et al A composite of multiple signals distinguishes causal variants in regions of positive selection. Science 2010, 327:883–6. 10.1126/science.1183863 20056855

[74] Mizuno H, Atwal G, Wang H, Levine A, Vazquez A. Fine-scale detection of population-specific linkage disequilibrium using haplotype entropy in the human genome. BMC Genet 2010, 11:27 10.1186/1471-2156-11-27 20416085 PMC2873552

[75] Cai Z, Camp NJ, Cannon-Albright L, Thomas A. Identification of regions of positive selection using shared genomic segment analysis. Eur J Hum Genet 2011, 19:667–671. 10.1038/ejhg.2010.257 21304558 PMC3110045

[76] Grossman SR, Andersen KG, Shlyakhter I, Tabrizi S, Winnicki S, Yen A, et al Identifying recent adaptations in large-scale genomic data. Cell 2013, 152:703–713. 10.1016/j.cell.2013.01.035 23415221 PMC3674781

[77] Liu X, Ong RT-H, Pillai EN, Elzein AM, Small KS, Clark TG, et al Detecting and characterizing genomic signatures of positive selection in global populations. Am J Hum Genet 2013, 92:866–881. 10.1016/j.ajhg.2013.04.021 23731540 PMC3675259

[78] Fagny M, Patin E, Enard D, Barreiro LB, Quintana-Murci L, Laval G. Exploring the occurrence of classic selective sweeps in humans using whole-genome sequencing data sets. Mol Biol Evol 2014, 31:1850–1868. 10.1093/molbev/msu118 24694833

[79] Haasl RJ, Johnson RC, Payseur BA. The effects of microsatellite selection on linked sequence diversity. Genome Biol Evol 2014, 6:1843–1861. 25115009 10.1093/gbe/evu134 PMC4122932

[80] Rafajlović M, Klassmann A, Eriksson A, Wiehe T, Mehlig B. Demography-adjusted tests of neutrality based on genome-wide SNP data. Theor Popul Biol 2014, 95:1–12. 10.1016/j.tpb.2014.05.002 24911258

[81] Venny. An interactive tool for comparing lists with Venn’s diagrams [http://bioinfogp.cnb.csic.es/tools/venny/index.html]

[82] Roy PJ, Stuart JM, Lund J, Kim SK. Chromosomal clustering of muscle-expressed genes in *Caenorhabditis elegans* . Nature 2002, 418:975–979. 12214599 10.1038/nature01012

[83] Harrow J, Frankish A, Gonzalez JM, Tapanari E, Diekhans M, Kokocinski F, et al The reference human genome annotation for The ENCODE Project. Genome Res 2012, 22:1760–1774. 10.1101/gr.135350.111 22955987 PMC3431492

[84] Thomas PD, Campbell MJ, Kejariwal A, Mi H, Karlak B, Daverman R, et al PANTHER: A library of protein families and subfamilies indexed by function. Genome Res 2003, 13:2129–2141. 12952881 10.1101/gr.772403 PMC403709

[85] Bednarek AK, Keck-Waggoner CL, Daniel RL, Laflin KJ, Bergsagel PL, Kiguchi K, et al WWOX, the FRA16D gene, behaves as a suppressor of tumor growth. Cancer Res 2001, 61:8068–8073. 11719429

[86] Chang N-S, Pratt N, Heath J, Schultz L, Sleve D, Carey GB, et al Hyaluronidase induction of a WW domain-containing oxidoreductase that enhances tumor necrosis factor cytotoxicity. J Biol Chem 2001, 276:3361–3370. 11058590 10.1074/jbc.M007140200

[87] Gogat K, Le Gat L, Van Den Berghe L, Marchant D, Kobetz A, Gadin S, et al, 2004 VEGF and KDR gene expression during human embryonic and fetal eye development. Invest Ophthalmol Vis Sci 2004, 45:7–14. 14691147 10.1167/iovs.02-1096

[88] Sawamiphak S, Seidel S, Essmann CL, Wilkinson GA, Pitulescu ME, Acker T, et al Ephrin-B2 regulates VEGFR2 function in developmental and tumour angiogenesis. Nature 2010, 465:487–491. 10.1038/nature08995 20445540

[89] Matsumoto M, Nakagawa T, Inoue T, Nagata E, Tanaka K, Takano H, et al Ataxia and epileptic seizures in mice lacking type 1 inositol 1,4,5-trisphosphate receptor. Nature 1996, 379:168–171. 8538767 10.1038/379168a0

[90] Wang Y, Li G, Goode J, Paz JC, Ouyang K, Screaton R, et al Inositol-1,4,5-trisphosphate receptor regulates hepatic gluconeogenesis in fasting and diabetes. Nature 2012, 485:128–132. 10.1038/nature10988 22495310 PMC3343222

[91] Mallaret M, Synofzik M, Lee J, Sagum CA, Mahajnah M, Sharkia R, et al The tumour suppressor gene WWOX is mutated in autosomal recessive cerebellar ataxia with epilepsy and mental retardation. Brain 2014, 137:411–419. 10.1093/brain/awt338 24369382 PMC3914474

[92] Abdel-Salam G, Thoenes M, Afifi H, Korber F, Swan D, Bolz H. The supposed tumor suppressor gene WWOX is mutated in an early lethal microcephaly syndrome with epilepsy, growth retardation and retinal degeneration. Orphanet J Rare Dis 2014, 9:12 10.1186/1750-1172-9-12 24456803 PMC3918143

[93] Mignot C, Lambert L, Pasquier L, Bienvenu T, Delahaye-Duriez A, Keren B, et al WWOX-related encephalopathies. delineation of the phenotypical spectrum and emerging genotype-phenotype correlation. J Med Genet 2015, 52:61–70. 10.1136/jmedgenet-2014-102748 25411445

[94] Synofzik M, Beetz C, Bauer C, Bonin M, Sanchez-Ferrero E, Schmitz-Hübsch T, et al Spinocerebellar ataxia type 15. diagnostic assessment, frequency, and phenotypic features. J Med Genet 2011, 48:407–412. 10.1136/jmg.2010.087023 21367767

[95] Iwaki A, Kawano Y, Miura S, Shibata H, Matsuse D, Li W, et al Heterozygous deletion of ITPR1, but not SUMF1, in spinocerebellar ataxia type 16. J Med Genet 2008, 45:32–35. 17932120 10.1136/jmg.2007.053942

[96] Su M-T, Lin S-H, Lee I-W, Chen Y-C, Kuo P-L. Association of polymorphisms/haplotypes of the genes encoding vascular endothelial growth factor and its KDR receptor with recurrent pregnancy loss. Hum Reprod 2011, 26:758–764. 10.1093/humrep/deq401 21257617

[97] Di Rienzo A. Population genetics models of common diseases. Genomes Evol 2006, 16:630–636. 10.1016/j.gde.2006.10.002 17055247

[98] Behrman et al Preterm Birth Causes, Consequences, and Prevention. Washington, DC The National Academies Press; 2007. 20669423

[99] Haygood R, Babbitt CC, Fedrigo O, Wray GA. Contrasts between adaptive coding and noncoding changes during human evolution. Proc Natl Acad Sci 2010, 107:7853–7857. 10.1073/pnas.0911249107 20385805 PMC2867918

[100] Oda K, Matsuoka Y, Funahashi A, Kitano H. A comprehensive pathway map of epidermal growth factor receptor signaling. Mol Syst Biol 2005, 1:2005.0010–2005.0010. 10.1038/msb4100014 PMC1681468 16729045

[101] Hoch RV, Soriano P. Roles of PDGF in animal development. Development 2003, 130:4769–4784. 12952899 10.1242/dev.00721

[102] Hakonen E, Ustinov J, Palgi J, Miettinen PJ, Otonkoski T. EGFR signaling promotes β-cell proliferation and survivin expression during pregnancy. PLoS ONE 2014, 9:e93651 10.1371/journal.pone.0093651 24695557 PMC3973552

[103] Nilsson UW, Johns TG, Wilmann T, Kaitu’u-Lino T, Whitehead C, Dimitriadis E, et al Effects of gefitinib, an epidermal growth factor receptor inhibitor, on human placental cell Growth. Obstet Gynecol 2013, 122. 10.1097/AOG.0b013e3182a1ba56 24084529

[104] Chhabra A, Lechner AJ, Ueno M, Acharya A, Van Handel B, Wang Y, et al Trophoblasts regulate the placental hematopoietic niche through PDGF-B signaling. Dev Cell 2012, 22:651–659. 10.1016/j.devcel.2011.12.022 22387002 PMC3395466

[105] Goustin AS, Betsholtz C, Pfeifer-Ohlsson S, Persson H, Rydnert J, Bywater M, et al Coexpression of the sis and myc proto-oncogenes in developing human placenta suggests autocrine control of trophoblast growth. Cell 1985, 41:301–312. 2986848 10.1016/0092-8674(85)90083-2

[106] Logan CY, Nusse R. The wnt signaling pathway in development and disease. Annu Rev Cell Dev Biol 2004, 20:781–810. 15473860 10.1146/annurev.cellbio.20.010403.113126

[107] Chen Q, Zhang Y, Lu J, Wang Q, Wang S, Cao Y, et al Embryo–uterine cross-talk during implantation. the role of Wnt signaling. Mol Hum Reprod 2009, 15:215–221. 10.1093/molehr/gap009 19223336

[108] Knöfler M, Pollheimer J. Human placental trophoblast invasion and differentiation: A particular focus on Wnt signaling. Front Genet 2013, 4:190 10.3389/fgene.2013.00190 24133501 PMC3783976

[109] Kim S-Y, Yasuda S, Tanaka H, Yamagata K, Kim H. Non-clustered protocadherin. Cell Adhes Migr 2011, 5:97–105. 10.4161/cam.5.2.14374 PMC3084973 21173574

[110] Redies C, Neudert F, Lin J. Cadherins in cerebellar development. translation of embryonic patterning into mature functional compartmentalization. The Cerebellum 2011, 10:393–408. 10.1007/s12311-010-0207-4 20820976

[111] Hirano S, Takeichi M. Cadherins in brain morphogenesis and wiring. Physiol Rev 2012, 92:597–634. 10.1152/physrev.00014.2011 22535893

[112] Chen WV, Maniatis T. Clustered protocadherins. Dev Camb Engl 2013, 140:3297–3302. 10.1242/dev.090621 PMC3737714 23900538

[113] Paulson AF, Prasad MS, Thuringer AH, Manzerra P. Regulation of cadherin expression in nervous system development. Cell Adhes Migr 2014, 8:19–28. 10.4161/cam.27839 PMC3974789 24526207

[114] Howard S, Deroo T, Fujita Y, Itasaki N. A positive role of cadherin in wnt/β-catenin signalling during epithelial-mesenchymal transition. PLoS ONE 2011, 6:e23899 10.1371/journal.pone.0023899 21909376 PMC3166074

[115] Heuberger J, Birchmeier W. Interplay of cadherin-mediated cell adhesion and canonical wnt signaling. Cold Spring Harb Perspect Biol 2010, 2:a002915 10.1101/cshperspect.a002915 20182623 PMC2828280

[116] Nelson WJ, Nusse R. Convergence of wnt, β-catenin, and cadherin pathways. Science 2004, 303:1483–1487. 15001769 10.1126/science.1094291 PMC3372896

[117] Marie PJ, Hay E. Cadherins and Wnt signalling. a functional link controlling bone formation. BoneKEy Rep 2013, 2. 10.1038/bonekey.2013.64 PMC3722765 24422077

[118] Schwenke M, Knöfler M, Velicky P, Weimar CHE, Kruse M, et al Control of human endometrial stromal cell motility by PDGF-BB, HB-EGF and trophoblast-secreted factors. PLoS ONE 2013, 8:e54336 10.1371/journal.pone.0054336 23349855 PMC3549986

[119] Large MJ, Wetendorf M, Lanz RB, Hartig SM, Creighton CJ, Mancini MA, et al The epidermal growth factor receptor critically regulates endometrial function during early pregnancy. PLoS Genet 2014, 10:e1004451 10.1371/journal.pgen.1004451 24945252 PMC4063709

[120] Faxen M, Nasiell J, Black A, Nisell H, Lunell N. Altered mRNA expression pattern of placental epidermal growth factor receptor (EGFR) in pregnancies complicated by preeclampsia and/or intrauterine growth retardation. Amer J Perinatol, 15:9–13. 9475680 10.1055/s-2007-993890

[121] Fondacci C, Alsat E, Gabriel R, Blot P, Nessmann C, Evain-Brion D. Alterations of human placental epidermal growth factor receptor in intrauterine growth retardation. J Clin Invest 1994, 93:1149–1155. 8132754 10.1172/JCI117067 PMC294064

[122] Dissanayake VHW, Tower C, Broderick A, Stocker LJ, Seneviratne HR, Jayasekara RW, et al Polymorphism in the epidermal growth factor gene is associated with birthweight in Sinhalese and white Western Europeans. Mol Hum Reprod 2007, 13:425–429. 17392355 10.1093/molehr/gam011

[123] Jurcčovicčová J, Krueger KS, Nandy I, Lewis DF, Brooks GG, Brown EG. Expression of platelet-derived growth factor-A mRNA in human placenta. Effect of magnesium infusion in pre-eclampsia. Placenta, 19:423–427. 9699964 10.1016/s0143-4004(98)90083-2

[124] Bao SH, Shuai W, Tong J, Wang L, Chen P, Duan T. Increased Dickkopf-1 expression in patients with unexplained recurrent spontaneous miscarriage. Clin Exp Immunol 2013, 172:437–443. 10.1111/cei.12066 23600832 PMC3646443

[125] Zhang Z, Li H, Zhang L, Jia L, Wang P. Differential expression of beta-catenin and dickkopf-1 in the third trimester placentas from normal and preeclamptic pregnancies. a comparative study. Reprod Biol Endocrinol 2013, 11:17 10.1186/1477-7827-11-17 23452984 PMC3599361

[126] Fritz R, Jain C, Armant R. Cell signaling in trophoblast-uterine communication. Int J Dev Biol 2014, 58:261–271. 10.1387/ijdb.140011da 25023692 PMC10411524

[127] Lindsay D, Poulson E, Robson JM. The effect of 5-Hydroxytryptamine on pregnancy. J Endocrinol 1963, 26:85–96. 13930825 10.1677/joe.0.0260085

[128] Kelly CR, Sharif NA. Pharmacological evidence for a functional serotonin-2B receptor in a human uterine smooth muscle cell line. J Pharmacol Exp Ther 2006, 317:1254–1261. 16517693 10.1124/jpet.105.100172

[129] Cordeaux Y, Pasupathy D, Bacon J, Charnock-Jones DS, Smith GCS. Characterization of serotonin receptors in pregnant human myometrium. J Pharmacol Exp Ther 2009, 328:682–691. 10.1124/jpet.108.143040 19075042

[130] Kitazawa T, Kubo O, Satoh M, Taneike T. Involvement of 5-hydroxytryptamine7 receptors in inhibition of porcine myometrial contractility by 5-hydroxytryptamine. Br J Pharmacol 1998, 123:173–182. 9489604 10.1038/sj.bjp.0701583 PMC1565149

[131] Jones RL, Stoikos C, Findlay JK, Salamonsen LA. TGF-β superfamily expression and actions in the endometrium and placenta. Reproduction 2006, 132:217–232. 16885531 10.1530/rep.1.01076

[132] Powe CE, Levine RJ, Karumanchi SA. Preeclampsia, a disease of the maternal endothelium. the role of anti-angiogenic factors and implications for later cardiovascular disease. Circulation 2011, 123: 10.1161/CIRCULATIONAHA.109.853127 PMC3148781 21690502

[133] Li Q. Transforming growth factor β signaling in uterine development and function. J Anim Sci Biotechnol 2014, 5:1–9. 25478164 10.1186/2049-1891-5-52 PMC4255921

[134] Brosens I, Pijnenborg R, Vercruysse L, Romero R. The “Great Obstetrical Syndromes” are associated with disorders of deep placentation. Am J Obstet Gynecol 2011, 204:193–201. 10.1016/j.ajog.2010.08.009 21094932 PMC3369813

[135] Carter AM, Pijnenborg R. Evolution of invasive placentation with special reference to non-human primates. Best Pr Res Clin Obstet Gynaecol 2011, 25:249–57. 10.1016/j.bpobgyn.2010.10.010 21056010

[136] Elliot MG, Crespi BJ. Genetic recapitulation of human pre-eclampsia risk during convergent evolution of reduced placental invasiveness in eutherian mammals. Philos Trans R Soc Lond B Biol Sci 2015, 370. 10.1098/rstb.2014.0069 PMC4305170 25602073

[137] Dunsworth HM, Warrener AG, Deacon T, Ellison PT, Pontzer H. Metabolic hypothesis for human altriciality. Proc Natl Acad Sci 2012, 109:15212–15216. 22932870 10.1073/pnas.1205282109 PMC3458333

[138] Wells JCK. Between Scylla and Charybdis. renegotiating resolution of the “obstetric dilemma” in response to ecological change. Philos Trans R Soc Lond B Biol Sci 2015, 370. 10.1098/rstb.2014.0067 PMC4305168 25602071

[139] Rockwell LC, Vargas E, Moore LG. Human physiological adaptation to pregnancy. Inter- and intraspecific perspectives. Am J Hum Biol 2003, 15:330–341. 12704709 10.1002/ajhb.10151

[140] Browne VA, Julian CG, Toledo-Jaldin L, Cioffi-Ragan D, Vargas E, Moore LG. Uterine artery blood flow, fetal hypoxia and fetal growth. Philos Trans R Soc Lond B Biol Sci 2015, 370. 10.1098/rstb.2014.0068 PMC4305169 25602072

[141] Moffett A, Hiby SE, Sharkey AM. The role of the maternal immune system in the regulation of human birthweight. Philos Trans R Soc Lond B Biol Sci 2015, 370. 10.1098/rstb.2014.0071 PMC4305172 25602075

[142] Weiner S, Monge J, Mann A. Bipedalism and parturition. an evolutionary imperative for cesarean delivery? Cesarean Deliv Its Impact Mother Newborn Part II 2008, 35:469–478. 10.1016/j.clp.2008.06.003 18952015

[143] Jukic A, Baird D, Weinberg C, McConnaughey D, Wilcox A. Length of human pregnancy and contributors to its natural variation. Hum Reprod Oxf Engl 2013, 28:2848–2855. 10.1093/humrep/det297 PMC3777570 23922246

[144] Kieler H, Axelsson O, Nilsson S, Waldenströ U. The length of human pregnancy as calculated by ultrasonographic measurement of the fetal biparietal diameter. Ultrasound Obstet Gynecol 1995, 6:353–357. 8590208 10.1046/j.1469-0705.1995.06050353.x

[145] Nassar N, Schiff M, Roberts CL. Trends in the distribution of gestational age and contribution of planned births in New South Wales, Australia. PLoS ONE 2013, 8:e56238 10.1371/journal.pone.0056238 23437101 PMC3577819

[146] Phillips JB, Abbot P, Rokas A. Is preterm birth a human-specific syndrome? Evol Med Public Health 2015. 10.1093/emph/eov010 PMC4493222 26077822

[147] Ozcelik T, Akarsu N, Uz E, Caglayan S, Gulsuner S, Onat OE, et al Mutations in the very low-density lipoprotein receptor VLDLR cause cerebellar hypoplasia and quadrupedal locomotion in humans. Proc Natl Acad Sci 2008, 105:4232–4236. 10.1073/pnas.0710010105 18326629 PMC2393756

[148] Dorus S, Vallender EJ, Evans PD, Anderson JR, Gilbert SL, Mahowald M, et al Accelerated evolution of nervous system genes in the origin of *Homo sapiens* . Cell 2004, 119:1027–1040. 15620360 10.1016/j.cell.2004.11.040

[149] Shi P, Bakewell MA, Zhang J. Did brain-specific genes evolve faster in humans than in chimpanzees? Trends Genet 2006, 22:608–613. 16978728 10.1016/j.tig.2006.09.001

[150] Haygood R, Fedrigo O, Hanson B, Yokoyama K-D, Wray GA. Promoter regions of many neural- and nutrition-related genes have experienced positive selection during human evolution. Nat Genet 2007, 39:1140–1144. 17694055 10.1038/ng2104

[151] Zhang YE, Landback P, Vibranovski MD, Long M. Accelerated recruitment of new brain development genes into the human genome. PLoS Biol 2011, 9:e1001179 10.1371/journal.pbio.1001179 22028629 PMC3196496

[152] Popesco MC, MacLaren EJ, Hopkins J, Dumas L, Cox M, Meltesen L, et al Human lineage-specific amplification, selection, and neuronal expression of DUF1220 domains. Science 2006, 313:1304–1307. 16946073 10.1126/science.1127980

[153] Brunetti-Pierri N, Berg JS, Scaglia F, Belmont J, Bacino CA, Sahoo T, et al Recurrent reciprocal 1q21.1 deletions and duplications associated with microcephaly or macrocephaly and developmental and behavioral abnormalities. Nat Genet 2008, 40:1466–1471. 10.1038/ng.279 19029900 PMC2680128

[154] Mefford HC, Sharp AJ, Baker C, Itsara A, Jiang Z, Buysse K, et al Recurrent rearrangements of chromosome 1q21.1 and variable pediatric phenotypes. N Engl J Med 2008, 359:1685–1699. 10.1056/NEJMoa0805384 18784092 PMC2703742

[155] Pinto D, Pagnamenta AT, Klei L, Anney R, Merico D, Regan R, et al Functional impact of global rare copy number variation in autism spectrum disorders. Nature 2010, 466:368–372. 10.1038/nature09146 20531469 PMC3021798

[156] Levinson DF, Duan J, Oh S, Wang K, Sanders AR, Shi J, et al Copy number variants in schizophrenia. confirmation of five previous findings and new evidence for 3q29 microdeletions and VIPR2 duplications. Am J Psychiatry 2011, 168:302–316. 10.1176/appi.ajp.2010.10060876 21285140 PMC4441324

[157] Dumas LJ, O’Bleness MS, Davis JM, Dickens CM, Anderson N, Keeney J, et al DUF1220-domain copy number implicated in human brain-size pathology and evolution. Am J Hum Genet 2012, 91:444–454. 10.1016/j.ajhg.2012.07.016 22901949 PMC3511999

[158] Davis JM, Searles VB, Anderson N, Keeney J, Dumas L, Sikela JM. DUF1220 dosage is linearly associated with increasing severity of the three primary symptoms of autism. PLoS Genet 2014, 10:e1004241 10.1371/journal.pgen.1004241 24651471 PMC3961203

[159] Keeney JG, Dumas L, Sikela JM. The case for DUF1220 domain dosage as a primary contributor to anthropoid brain expansion. Front Hum Neurosci 2014, 8:427 10.3389/fnhum.2014.00427 25009482 PMC4067907

[160] Anum EA, Springel EH, Shriver MD, Strauss JF 3rd. Genetic contributions to disparities in preterm birth. Pediatr Res 2009, 65:1–9. 10.1203/PDR.0b013e31818912e7 18787421 PMC2651992

