# Supplementary material for: Integrating Diverse Types of Genomic Data to Identify Genes that Underlie Adverse Pregnancy Phenotypes
Source: PLoS One. 2015 Dec 7;10(12):e0144155. doi: 10.1371/journal.pone.0144155 (PMC4671692; doi:10.1371/journal.pone.0144155)
Supplement: S1 Materials — (DOCX) [file pone.0144155.s001.docx]

**S1 Materials. Additional methods and rationale for detecting overlap of regions and genes exhibiting accelerated evolutionary rates.**

Several methods that use site frequencies or haplotype patterns have been developed to detect selective events from patterns of genetic variation. The power of all these methods to detect signatures of adaptive changes depend on the level of sequence coverage and SNP density, SNP ascertainment (in case of SNP data like HapMap), intensity of selection, admixture and recombination rate estimates [1–3]. Error and/or uncertainty in these parameters when interrogating the human population for signature of evolution will bias the results and inferences made from such analyses [3]. In fact, several studies have reviewed overlap of the human genes/genomic regions inferred to bear signatures of positive selection using these different methods and showed only modest overlap and replication [2, 4–8], despite the expectations that there should be substantial overlap between different studies. Most authors attribute the low levels of replication to several factors, including coverage depth, uncertainty in demographic patterns, uncertainty in recombination rates and differences in power between the methods to detect different kind of selection [2, 4–8].

Previous comparative studies did not differentiate between the kinds of data (HapMap or Perlegen) and populations used to infer overlap [2, 4–8]. These two aspects are important considering the fact that low coverage data and SNP typing such as HapMap data with ascertainment bias limit the ability of these statistical methods to detect signatures of adaptive evolution. Moreover, signatures of positive selection are not uniformly distributed across populations, but conform to patterns of local adaptation [7]. However, comparison of results with the same methods and populations but different kinds of data, and those with same kinds of data and populations but using different methods, exhibit similar low albeit statistically significant overlap [2, 4, 6, 7].

In human population data, there is statistically significant overlap between regions/genes that are inferred using different methods to be under signature of fast evolution even when results from different public data for European population are considered **(S6 Table)**. For example, data from 1000 Genome Pilot Phase data had no overlap with nearly 900 genes identified in CEU populations. Moreover, data using the same haplotype method on (iHS) European populations but different data types also showed limited overlap; only one gene that is shared across all the data types. Despite this modest overlap between studies, the loci that have been identified in these different studies represent genuine signatures of adaptive evolution, and might actually “represent the most dramatic selective events” [7]. Moreover, the methods commonly used to detect signature of adaptive evolution have been evaluated by simulation and shown to be robust to underlying assumptions [2]. These results validate inferences in this study regarding accelerated gene regions and the likelihood of positive selection.

For fast evolution in coding sections of the genome, we collated data from 11 studies that looked at primate genomes to infer positive selection using variations of McDonald Kreitman test (dN/dS) [9–20]. For each study, we selected genes that met specific significance criteria used by each study to infer positive selection (after multiple corrections). The genes identified by different studies showed limited overlap, likely due to differences in methodology, such as the manner in which different data were generated and filtered [20]. Moreover, the same kinds of issues that hamper methods used to detect signatures of adaptive changes in global human population, such as the level of sequence coverage, intensity of selection, admixture and recombination rate estimates, will affect the results and inferences made in such analyses. Ultimately, we collated a total of 1036 genes from these 11 studies into our category of Coding Adaptive Changes **(S1 Table)**. Below, we speculate on some interpretations of genes detected by these measures and their patterns of overlap.

|  | Evolutionary interpretation of genes that are identified by… |
| --- | --- |
| HARs only | Positive selection in functional elements of genes in the human lineage, often associated with developmental genes, transcription factors, and genes expressed in the central nervous system. Typically, older substitution shared by all human populations. |
| EPS only | Positive selection largely in non-coding regions of genes of European ancestry. Typically, more recently derived and population (European) specific. |
| CAC only | Positive selection in or near exonic regions of human genes, typically, older substitutions shared by all human populations. |
| HARS + EPS | Consensus on substitutions affecting gene regulation |
| HARS + CAC | Positive selection in or near coding and non-coding gene regions. Likely more ancient, emerging early in the human lineage |
| EPS + CAC | Positive selection in or near coding and non-coding gene regions, in which non-coding segregations may signify local adaptation in gene expression |
| HARS, EPS, CAC | Positive selection in or near coding and non-coding gene regions, both older and more recent. Of particular interest. |

**Supplementary Materials and Tables Legends**

**S1 Materials. Additional methods and rationale for detecting overlap of regions and genes exhibiting accelerated evolutionary rates.**

**S1 Table. Lists of genes used for all of the analyses described.**

**S2 Table. Panther categorization of the 12,248 placental expressed genes and their overlap with fast evolving genes.**

**S3 Table.** **Panther categorization of the placental enriched genes from the Protein Atlas Database and their overlap with fast evolving genes.**

**S4 Table.** **Panther categorization of the genes differentially expressed in four PTB clinical subtypes (collated by Eidem et al. 2015) and their overlap with fast evolving genes.** There were no overrepresented fast evolving genes in any category, and thus these tables are not shown.

**S5 Table: Summary of selected genes that exhibit the overlap across multiple categories of fast evolution and expression or disease phenotypes, and synopses of Entrez gene summaries and associated phenotypes.** See **S1** Table for the full list of overlapping genes.

**S6 Table A**: **Overlap between genes that fall in genomic regions that are fast evolving in European populations based on different selection methods on 1000 Genomes project data.** There is proportionally more overlap between data from STR method and site frequency spectrum. **B**: Overlap between fast evolving genes in European populations based on integrated haplotype scores (iHS) methods on four different data type of European populations. There is proportionally more overlap between genes inferred from HapMapII and human genome diversity project data than any other pairwise comparison. Statistical significance of the overlap between genes from different methods inferred using hypergeometric method as implemented in <http://nemates.org/MA/progs/overlap_stats.html>.

***References***

1. Sabeti PC, Varilly P, Fry B, Lohmueller J, Hostetter E, Cotsapas C, et al.: Genome-wide detection and characterization of positive selection in human populations. Nature 2007, 449:913–918.

2. Nielsen R, Hellmann I, Hubisz M, Bustamante C, Clark AG: Recent and ongoing selection in the human genome. Nat Rev Genet 2007, 8:857–868.

3. Scheinfeldt LB, Tishkoff SA: Recent human adaptation: genomic approaches, interpretation and insights. Nat Rev Genet 2013, 14:692–702.

4. Biswas S, Akey JM: Genomic insights into positive selection. Trends Genet 2006, 22:437–446.

5. Oleksyk TK, Zhao K, De La Vega FM, Gilbert DA, O’Brien SJ, Smith MW. Identifying selected regions from heterozygosity and divergence using a light-coverage genomic dataset from two human populations. PLoS ONE 2008, 3:e1712.

6. Oleksyk TK, Smith MW, O’Brien SJ: Genome-wide scans for footprints of natural selection. Philos Trans R Soc B Biol Sci 2010, 365:185–205.

7. Akey JM: Constructing genomic maps of positive selection in humans: Where do we go from here?. Genome Res 2009, 19:711–722.

8. Pritchard JK, Pickrell JK, Coop G: The genetics of human adaptation: hard sweeps, soft sweeps, and polygenic adaptation. Curr Biol CB 2010, 20:R208–R215.

9. Plunkett J, Doniger S, Orabona G, Morgan T, Haataja R, Hallman M, et al. An evolutionary genomic approach to identify genes involved in human birth timing. PLoS Genet 2011, 7:e1001365.

10. Bustamante CD, Fledel-Alon A, Williamson S, Nielsen R, Todd Hubisz M, Glanowski S, et al. Natural selection on protein-coding genes in the human genome. Nature 2005, 437:1153–1157.

11. Mikkelsen TS, Hillier LW, Eichler EE, Zody MC, Jaffe DB, Yang S-P, et al. Initial sequence of the chimpanzee genome and comparison with the human genome. Nature 2005, 437:69–87.

12. Bakewell MA, Shi P, Zhang J: More genes underwent positive selection in chimpanzee evolution than in human evolution. Proc Natl Acad Sci 2007, 104:7489–7494.

13. Gibbs RA, Rogers J, Katze MG, Bumgarner R, Weinstock GM, Mardis ER, et al.: Evolutionary and biomedical insights from the rhesus macaque genome. Science 2007, 316:222–234.

14. Enard D, Depaulis F, Roest Crollius H: Human and non-human primate genomes share hotspots of positive selection. PLoS Genet 2010, 6:e1000840.

15. Crisci JL, Wong A, Good JM, Jensen JD: On characterizing adaptive events unique to modern humans. Genome Biol Evol 2011, 3:791–798.

16. Crosley EJ, Elliot MG, Christians JK, Crespi BJ: Placental invasion, preeclampsia risk and adaptive molecular evolution at the origin of the great apes: evidence from genome-wide analyses. Placenta 2013, 34:127–32.

17. Gaya-Vidal M, Alba M: Uncovering adaptive evolution in the human lineage. BMC Genomics 2014, 15:599.

18. Worley KC, Warren WC, Rogers J, Locke D, Muzny DM, Mardis ER, et al.: The common marmoset genome provides insight into primate biology and evolution. Nat Genet 2014, 46:850–857.

19. Nielsen R, Bustamante C, Clark AG, Glanowski S, Sackton TB, Hubisz MJ, et al. A scan for positively selected genes in the genomes of humans and chimpanzees. PLoS Biol 2005, 3:e170.

20. George RD, McVicker G, Diederich R, Ng SB, MacKenzie AP, Swanson WJ, et al. Trans genomic capture and sequencing of primate exomes reveals new targets of positive selection. Genome Res 2011, 21:1686–1694.

21. Petronella N, Drouin G: Gene conversions in the growth hormone gene family of primates: Stronger homogenizing effects in the Hominidae lineage. Genomics 2011, 98:173–181.

22. Torgerson DG, Boyko AR, Hernandez RD, Indap A, Hu X, White TJ, et al. Evolutionary processes acting on candidate cis-regulatory regions in humans inferred from patterns of polymorphism and divergence. PLoS Genet 2009, 5:e1000592.

23. Lomelin D, Jorgenson E, Risch N: Human genetic variation recognizes functional elements in noncoding sequence. Genome Res 2010, 20:311–9.

24. Mu XJ, Lu ZJ, Kong Y, Lam HYK, Gerstein MB: Analysis of genomic variation in non-coding elements using population-scale sequencing data from the 1000 Genomes Project. Nucleic Acids Res 2011, 39:7058–7076.

25. Lindblad-Toh K, Garber M, Zuk O, Lin MF, Parker BJ, Washietl S, et al. A high-resolution map of human evolutionary constraint using 29 mammals. Nature 2011, 478:476–482.

26. Ward LD, Kellis M: Evidence of abundant purifying selection in humans for recently-acquired regulatory functions. Science 2012, 337:1675–1678.

27. King DC, Taylor J, Zhang Y, Cheng Y, Lawson HA, Martin J. et al. Finding cis-regulatory elements using comparative genomics: Some lessons from ENCODE data. Genome Res 2007, 17:775–786.

28. Lander ES, Schaffner SF, Sabeti PC, Grossman SR, Andersen KG, Shlyakhter I, et al. 1000 Genomes Project: Identifying recent adaptations in large-scale genomic data. Cell 2013, 152:703–713.

29. Durbin et al.: A map of human genome variation from population-scale sequencing. Nature 2010, 467:1061–1073.

30. Enard D, Messer PW, Petrov DA: Genome-wide signals of positive selection in human evolution. Genome Res 2014.

31. Fraser HB: Gene expression drives local adaptation in humans. Genome Res 2013, 23:1089–1096.

32. Wray GA: The evolutionary significance of cis-regulatory mutations. Nat Rev Genet 2007, 8:206–216.

33. Epstein DJ: Cis-regulatory mutations in human disease. Brief Funct Genomic Proteomic 2009, 8:310–316.

34. Capra JA, Erwin GD, McKinsey G, Rubenstein JLR, Pollard KS: Many human accelerated regions are developmental enhancers. Philos Trans R Soc Lond B Biol Sci 2013, 368.
