# Supplementary material for: Integrating Diverse Types of Genomic Data to Identify Genes that Underlie Adverse Pregnancy Phenotypes
Source: PLoS One. 2015 Dec 7;10(12):e0144155. doi: 10.1371/journal.pone.0144155 (PMC4671692; doi:10.1371/journal.pone.0144155)
Supplement: S5 Table — See Table for the full list of overlapping genes. (DOCX) [file pone.0144155.s006.docx]

**S5 Table: Summary of selected genes that exhibit the overlap across multiple categories of fast evolution and expression or disease phenotypes, and synopses of Entrez gene summaries and associated phenotypes.** See S1 Table for the full list of overlapping genes.

| **Gene** | **Name** | **Overlap** | **Function and phenotypes** |
| --- | --- | --- | --- |
| PARD3B | Partitioning defective 3 homolog B | PEG^1^, HARs, EPS | Encodes proteins involved in cell division and cell polarization processes, and formation of epithelial tight junctions; loss of expression promotes cell proliferation in some cancers. Neural tube defect, fetal structural abnormalities, and amyotrophic lateral sclerosis 2 |
| WWOX | WW domain containing oxidoreductase | PEG, HARs, EPS; also PE^2^, HARs, EPS | Encodes a member of the short-chain dehydrogenases/reductases (SDR) protein family, important in regulation of protein degradation, transcription, and RNA splicing. Spinocerebellar ataxia, early infantile epileptic encephalopathy 28 |
| NBPF11, 12, 15 |  | PEG, PE, sPTB, EPS, HARs | These genes are members of the Neuroblastoma Breakpoint Gene Family (NBPF), which contain multiple tandem repeated sequences termed DUF220 that have been shown to be associated with normal and pathological brain-size variations in humans, and a number of mainly brain related diseases. |
| IL1RL1 |  | PEG, LED, sPTB, EPS, HARs | Encodes a protein speculated to be involved in helper T-cell function, and implicated in the progression of cardiac disease |
| PAPPA PAPPA2 PAPPA-AS1 | Pappalysin-1 Pappalysin-2 | placentally enriched, CAC, HARs; also sPTB, HARs | Encodes a secreted metalloproteinase that cleaves insulin-like growth factor binding proteins, involved in local proliferative processes such as wound healing and bone remodeling. Intrauterine growth restriction, premature delivery, preeclampsia, and stillbirth. |
| ADAM12 | Disintegrin and metalloproteinase domain-containing protein 12 | placentally enriched, EPS | Involved in a variety of biological processes involving cell-cell and cell-matrix interactions, including fertilization, muscle development, and neurogenesis. Biomarker for prenatal development. |
| EPYC | Dermatan sulfate proteoglycan 3 | PEG, HARs, EPS, and placentally enriched, HARs, EPS | Leucine-rich repeat proteoglycan, regulates fibrillogenesis |
| HGF | Hepatocyte growth factor | placentally enriched, HARs, EPS | Regulates cell growth, motility, and morphogenesis; cytokine on cells of mainly epithelial origin; central role in angiogenesis, tumorogenesis, and tissue regeneration. |
| KDR | Vascular endothelial growth factor receptor 2 | LED^3^, HARs, EPS | Encodes receptors of vascular endothelial growth factors that mediate endothelial proliferation, survival, migration, tubular morphogenesis and sprouting. Associated diseases include recurrent pregnancy loss |
| ITPR1 | Inositol 1,4,5-trisphosphate receptor type 1 | LED, HARs, EPS | Encodes a receptor that mediates calcium release from the endoplasmic reticulum that triggers apoptosis. Spinocerebellar ataxia 15. |
| CFB | Complement factor B | PPROM^4^, CAC, EPS | Encodes complement factor B, a component of the alternative pathway of complement activation. Complement factor B deficiency and hemolytic uremic syndrome. |
| CXCR4 | C-X-C chemokine receptor type 4 | sPTB^5^, CAC, HARs, EPS | Encodes a CXC chemokine receptor specific for stromal cell-derived factor-1. Acts with the CD4 protein to support HIV entry into cells and is also highly expressed in breast cancer cells. WHIM syndrome and placental vascularization deficiency. |

**1. PEG = expressed in trophoblastic or decidual cells; 2. PE = preeclampsia; 3. LED = birth without labor; 4. PPROM = spontaneous rupture of membranes; 5. sPTB = spontaneous pre-term birth**
