# Supplementary material for: Integrating Diverse Types of Genomic Data to Identify Genes that Underlie Adverse Pregnancy Phenotypes
Source: PLoS One. 2015 Dec 7;10(12):e0144155. doi: 10.1371/journal.pone.0144155 (PMC4671692; doi:10.1371/journal.pone.0144155)
Supplement: S6 Table — Statistical significance of the overlap between genes from different methods inferred using hypergeometric method as implemented in http://nemates.org/MA/progs/overlap_stats.html. (DOCX) [file pone.0144155.s007.docx]

**S6 Table A**: **Overlap between genes that fall in genomic regions that are fast evolving in European populations based on different selection methods on 1000 Genomes project data.** There is proportionally more overlap between data from STR method and site frequency spectrum. **B**: Overlap between fast evolving genes in European populations based on integrated haplotype scores (iHS) methods on four different data type of European populations. There is proportionally more overlap between genes inferred from HapMapII and human genome diversity project data than any other pairwise comparison. Statistical significance of the overlap between genes from different methods inferred using hypergeometric method as implemented in <http://nemates.org/MA/progs/overlap_stats.html>.

**A:**

| **1000 genomes project** |  | **CMS** | | **iHS** | | **STR** | |
| --- | --- | --- | --- | --- | --- | --- | --- |
|  | **N** | **Overlap** | **p-value (RF)** | **Overlap** | **p-value (RF)** | **Overlap** | **p-value (RF)** |
| **CMS** | 164 |  |  |  |  |  |  |
| **iHS** | 230 | 14 | **<7.695e-09 (7.4)** |  |  |  |  |
| **STR** | 476 | 17 | **< 4.638e-07 (4.3)** | **16** | **< 1.488e-04 (2.9)** |  |  |
| **SFS** | 64 | 2 | ns | 0 | ns | 12 | **< 3.405e-08 (7.8)** |

**B:**

| **Europeans** |  | **1000 Genomes** | | **HapMapII** | | **Complete Genomics** | |
| --- | --- | --- | --- | --- | --- | --- | --- |
|  | **N** | **Overlap** | **p-value (RF)** | **Overlap** | **p-value (RF)** | **Overlap** | **p-value (RF)** |
| **1000 Genomes** | 230 |  |  |  |  |  |  |
| **HapMapII** | 80 | 8 | **< 4.124e-06 (8.6)** |  |  |  |  |
| **Complete Genomics** | 418 | 44 | **< 1.983e-29 (9.1)** | 5 | **< 0.027 (3.0)** |  |  |
| **HGDP** | 197 | 9 | **< 5.080e-04 (3.9)** | 14 | **< 4.916e-14 (17.6)** | 24 | **< 4.822e-12 (5.8)** |
